# Redshifted Cherenkov Radiation for *in vivo* Imaging: Coupling Cherenkov Radiation Energy Transfer to multiple Förster Resonance Energy Transfers

**DOI:** 10.1038/srep45063

**Published:** 2017-03-24

**Authors:** Yann Bernhard, Bertrand Collin, Richard A. Decréau

**Affiliations:** 1 Institut de Chimie Moléculaire, ICMUB CNRS UMR6302, University of Burgundy Franche-Comté, 9 avenue Alain Savary, 21078, Dijon, France; 2 Centre George-François Leclerc (CGFL), 1 rue du Professeur Marion, 21079, Dijon, France

## Abstract

Cherenkov Radiation (CR), this blue glow seen in nuclear reactors, is an optical light originating from energetic β-emitter radionuclides. CR emitter ^90^Y triggers a cascade of energy transfers in the presence of a mixed population of fluorophores (which each other match their respective absorption and emission maxima): Cherenkov Radiation Energy Transfer (CRET) first, followed by multiple Förster Resonance Energy transfers (FRET): CRET ratios were calculated to give a rough estimate of the transfer efficiency. While CR is blue-weighted (300–500 nm), such cascades of Energy Transfers allowed to get a) fluorescence emission up to 710 nm, which is beyond the main CR window and within the near-infrared (NIR) window where biological tissues are most transparent, b) to amplify this emission and boost the radiance on that window: EMT6-tumor bearing mice injected with both a radionuclide and a mixture of fluorophores having a good spectral overlap, were shown to have nearly a two-fold radiance boost (measured on a NIR window centered on the emission wavelength of the last fluorophore in the Energy Transfer cascade) compared to a tumor injected with the radionuclide only. Some CR embarked light source could be converted into a near-infrared radiation, where biological tissues are most transparent.

Cherenkov Radiation (CR) is the blue glow that may be seen in nuclear reactors (Fig. 1A)^1^,^2^,^3^. It arises during nuclear disintegration when emitted beta particles are energetic enough to move in a medium at a higher speed than light, and results in the emission of photons in the optical window^1^,^2^,^3^. Such a phenomenon, so-called the Vavilov-Cherenkov effect, is not only function of the energy of the emitted particle, but it also relies on the refractive index of the medium. CR is an optical radiation that quickly became of interest for medical imaging: Cherenkov Luminescence Imaging (CLI) is now used in preclinical^4^,^5^,^6^,^7^,^8^,^9^,^10^,^11^,^12^,^13^,^14^,^15^,^16^,^17^,^18^,^19^,^20^,^21^,^22^,^23^ and clinical^24^,^25^,^26^,^27^ settings. Some radiopharmaceuticals used in nuclear imaging (PET/SPECT) or radioimmunotherapy (RIT) are active in CLI, provided that they emit energetic enough beta particles^4^,^5^,^6^,^7^,^8^,^9^,^10^,^11^,^12^,^13^,^14^,^15^,^16^,^17^,^18^,^19^,^20^,^21^,^22^,^23^,^24^,^25^,^26^,^27^. However, the limitation of CR is that it barely penetrates tissues, because it is blue-weighted (300–600 nm) (Fig. 1B,C), whereas tissues are most transparent around 600–900 nm, i.e. the “therapeutic (optical) window” (window of tissue transparency)^28^. Hence, it appeared that an appropriate strategy might consist in transferring a portion of CR towards the near-infrared window by Cherenkov Radiation Energy Transfer (CRET). We, and others have demonstrated the feasibility of using CR to activate various platforms such as quantum dots and other nanoparticles, lanthanides, and fluorophores^14^,^15^,^16^,^17^,^18^,^19^,^20^,^21^,^22^,^23^. We rationalized the concept on organic fluorophores and showed that a single population of fluorophore, such as fluorescein, rhodamine-101 or rhodamine-6G, undergoes fluorescence emission upon irradiation by CR (from ^90^Y, ^177^Lu, or ^18^F)(Fig. 1B). Fluorescence emission (and high CRET ratios) was shown to be a function of the concentration of either the radionuclide or the fluorophore, and of the absorbance maxima that should match that of the CR peak emission (Fig. 1B) to get good CRET ratios^20^. Moreover, our earlier seminal studies were performed on single populations of fluorophores with modest Stokes shift (ca 20 nm)^20^. Hence, it prompted us to attempt to achieve ***larger transfers*** towards the NIR window (Fig. 1C), which will be described using more than a single population of fluorophores (Table 1). Such a goal will focus both on the extent of the overall (Stokes) *shifts* but also on the *ratio* of light being transferred: the overall goal of the study is a significant consumption of CR and subsequent transfer in the NIR window, a redshift of CR. Herein, this study describes the utilization of a technology relying on the combination of CRET and Förster Resonance Energy Transfers (FRET) processes that will be examined both on a spectrofluorimeter and an optical imager, and subsequently used to achieve Cherenkov Luminescence Imaging (CLI) *in vivo* on tumor-bearing mice in the NIR window.

## Spectrofluorimetric Studies

### CRET and CRET-FRET: *Stoke shifts*

**Figure 1:**
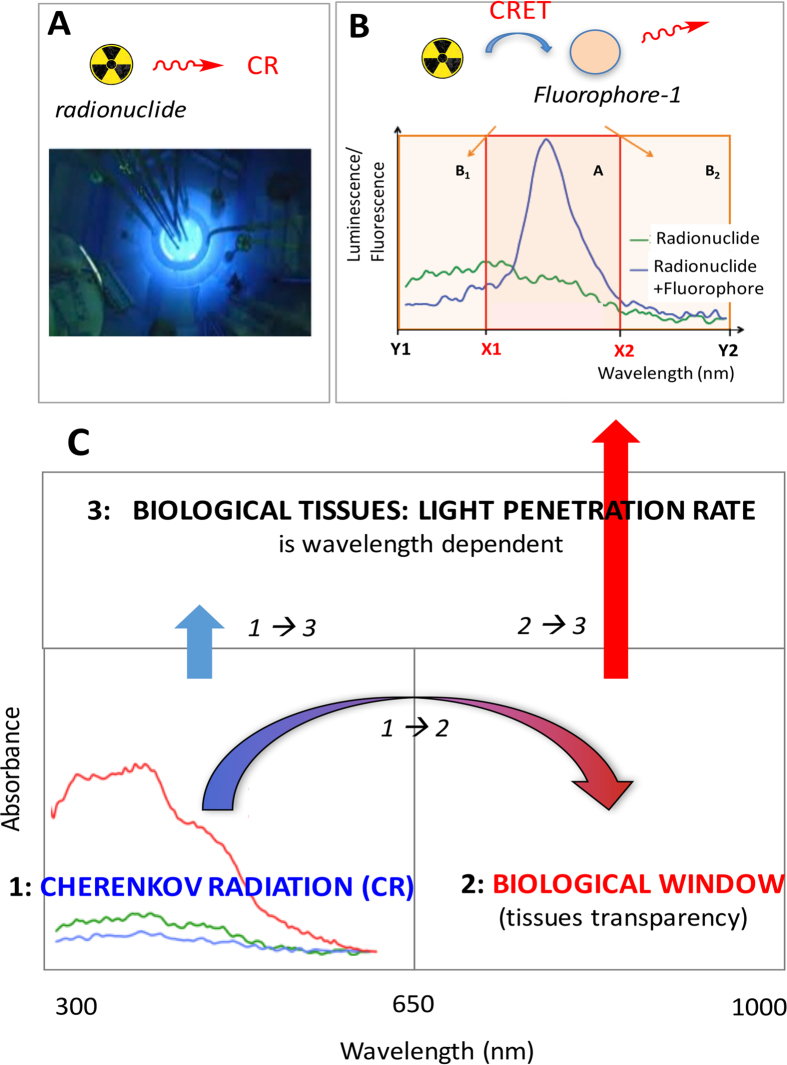
(**A**) Cherenkov Radiation (CR) as it appears in a nuclear reactor: emission of optical light that occurs from radionuclides. (**B**) CR-Energy Transfer (CRET): energy transfer of blue-weighted CR (optical spectrum represented in green line), from [^177^Lu]-LuCl_3_ (193 MBq) to fluorophore (fluorescein, 0.4 mM) that undergoes subsequent fluorescence emission (blue line)^20^. Windows A, B_1_, B_2_ are set for the measurement of CRET ratios (see methods). (**C**) purpose of the study: 1) to achieve the transfer of a significant portion of CR into the biologically relevant window, where tissues are most transparent (window of tissue transparency). As a result, light penetration through biological tissues will no longer be achieved from CR only (2) but also from light originating from the optical/biological window because of a mixture of fluorophores with good spectral overlap (3). Vertical arrows depict light penetration rate that is big in the NIR and small in the blue.

**Figure 2:**
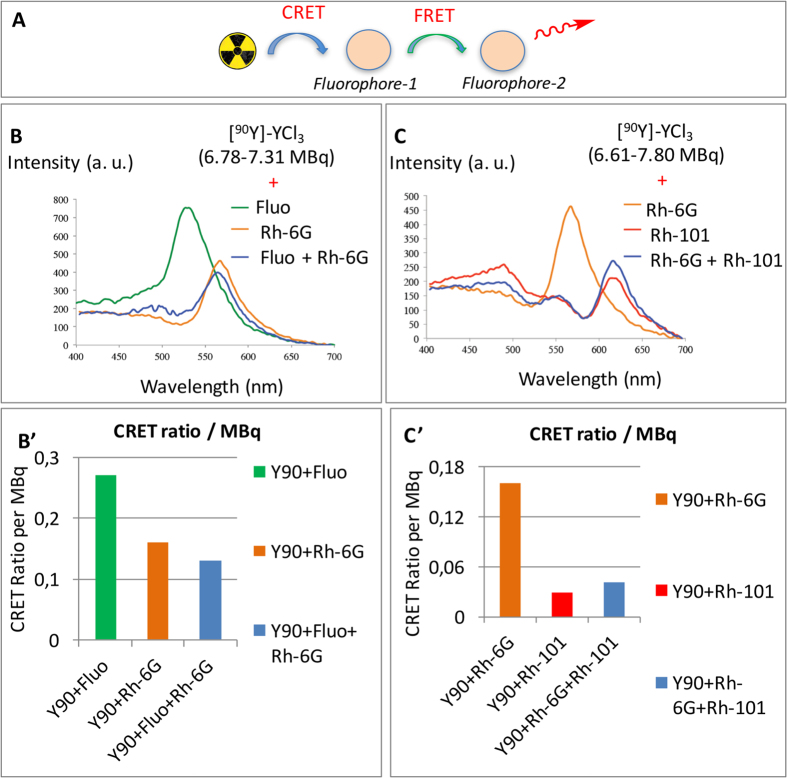
CRET-FRET transfers. (**A**) schematic representation. (**B**) overlapping of the fluorescence emission spectra of two individual fluorophores (with matching excitation/emission spectra) (CRET) and of a mixture of the two in the presence of ca. [^90^Y]-YCl_3_ (CRET-FRET). (**B**) fluoresceine alone (7.31 MBq [^90^Y]-YCl_3_, CRET), rhodamine-6G alone (6.78 MBq [^90^Y]-YCl_3_, CRET), and a mixture of both fluorescein + rhodamine-6G (6.89 MBq [^90^Y]-YCl_3_, CRET-FRET). B’: Bar graph addressing activity correcting spectral outputs (CRET ratios per activity). (**C**) rhodamine-6G alone (7.8 MBq [^90^Y]-YCl_3_, CRET), rhodamine-101 alone (6.61 MBq, CRET), and Rh-6G + Rh-101 mixture (7.80 MBq, CRET-FRET). C’: Bar graph addressing activity correcting spectral outputs (CRET ratios per activity). Concentration in fluorophore: 0.1 mM.

**Figure 3:**
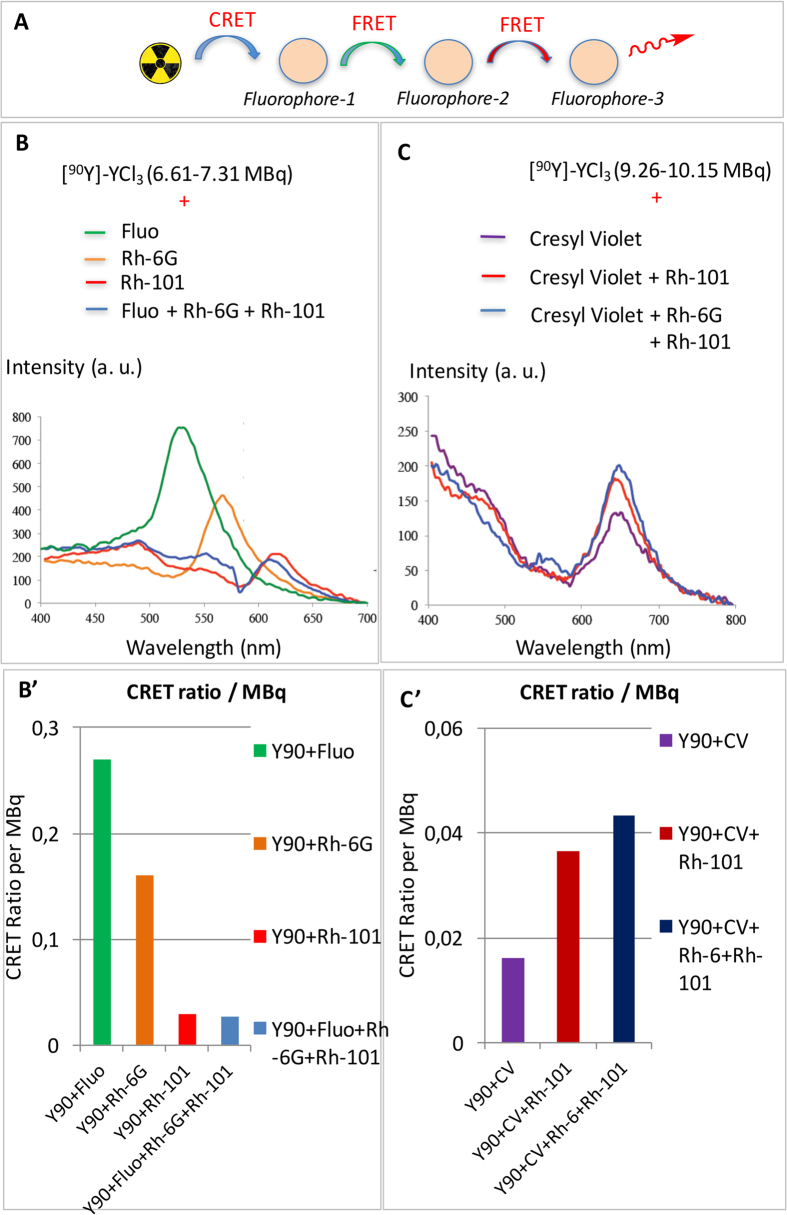
CRET-FRET-FRET. (**A**) schematic representation. (**B**) overlapping of the fluorescence emission spectra of three individual fluorophores (CRET) with matching excitation/emission spectra (fluorescein, rhodamine-6G, and rhodamine-101) and a mixture of the three fluorophores in the presence of [^90^Y]-YCl_3_ (6.61–7.31 MBq, CRET-FRET-FRET). Concentration in fluorophore 0.1 mM. B’: Bar graph addressing activity correcting spectral outputs (CRET ratios per activity). (**C**) overlapping of the fluorescence emission spectra of cresyl violet (CV) either alone (CRET), or mixed either with rhodamine-101 (CRET-FRET) or rhodamine-101 + rhodamine-6G (CRET-FRET-FRET) ([^90^Y]-YCl_3_ 9.26–10.15 MBq; concentration in fluorophore: 0.1 mM). Note the residual fluorescence peak of rhodamine-6G could be detected (small bump at 575 nm). C’: Bar graph addressing activity correcting spectral outputs (CRET ratios per activity).

Herein, in order to develop a strategy to transfer CR towards the near-IR window, a study was undertaken where the transfers occurred when two (Fig. 2A) or three (Fig. 3A) fluorophores were present in the cell, instead of one (Fig. 1B). The same set of fluorophores as that previously reported for CRET studies^20^ (Table 1) was selected because xanthenes are known to form efficient FRET pairs^29^. For instance binary systems that combine fluorescein and rhodamine (FITC/TRITC) are widely used in many areas. A careful examination of the emission spectrum of fluorescein (λ_ex_ 490 nm, λ_em_ 514 nm) and the absorption spectrum of rhodamine-6G (λ_ex_ 530 nm, λ_em_ 552 nm) shows a good spectral overlap between the two species. Also, Yttrium-90 was chosen as the source of radionuclide in our studies (in the form of [^90^Y]-YCl_3_) because it is a strong CR emitter: it emits energetic beta particle (2,200 kEv), which results in a high yield in photons (70 photons/disintegration)^11^. First, following our reported procedure^20^ the fluorescence emission spectra (Fig. 2B) of each individual fluorophore were revisited when CR was used as the excitation source (such as fluorescein (Fig. 2B, λ_em_ 514 nm, green line) and rhodamine-6G (Fig. 2B, λ_em_ 552 nm, orange line)). Next, when both fluorescein and rhodamine-6G were mixed together (in equimolar ratio) in the presence of a CR, the only fluorescence emission detected appears to be quite reminiscent of that of rhodamine-6G (λ_em_ 530 nm, Fig. 2B, blue line). No fluorescence emission from fluorescein (λ_em_ 514 nm) was observed. Hence, it suggests that if the captured CR photons at 490 nm do not give rise to an emission corresponding to that of fluoresceine, they are rather transferred to rhodamine-6G (λ_em_ 530–550 nm). Hence, it seems that the CRET process on fluorescein is then followed by a second transfer, Fluorescence Resonance Emission Transfer (FRET) to rhodamine-6G. Moreover, it is likely that rhodamine-6G is also activated by CR photons resulting in a CRET process, as previously indicated (Fig. 2A)^20^. Overall, fluorescein is subject to one energy transfer (CRET), whereas rhodamine-6G is subject to two transfers occurring simultaneously (CRET from ^90^Y and FRET from fluorescein). Such transfers correcting spectral outputs could also be represented as the form of CRET ratios per activity (Fig. 2B’, Table 2).

The same approach was carried out with rhodamine-6G (λ_ex_ 530 nm, λ_em_ 552 nm) and rhodamine-101 (λ_ex_ 576 nm, λ_em_ 600 nm): the emission spectrum of the former overlaps well with the absorption spectrum of the latter. After recording the fluorescence emission spectra of each individual fluorophore in the presence of CR (Fig. 2C: rhodamine-6G (514 nm, orange line) and rhodamine-101 (λ_em_ 600 nm, red line), fluorescence emission was recorded for a mixture containing both fluorophores (in equimolar ratio). A single emission band was detected, which corresponds to that of rhodamine-101 (Fig. 2C, blue line) and indicates a CRET-FRET process.

Then the transfers were examined when three fluorophores were present in the mixture (Fig. 3A–C), as opposed to two (Fig. 2A–C) or one (Fig. 1B). As stated above, there is a good overlap between the emission spectrum and absorption spectrum in the following two pairs of fluorophores: fluorescein/rhodamine-6G and rhodamine-6G/rhodamine-101 (Fig. 2B,C), as a result it is safe to state that there is a good spectral overlap for all three fluorophores. In a first example, when fluorescein, rhodamine-6G, and rhodamine-101 were mixed together with [^90^Y]-YCl_3_, only one emission band was observed at 600 nm that corresponds to that of rhodamine-101 (Fig. 3B). Such a result corresponds to a CRET-FRET-FRET process.

A second example of CRET-FRET-FRET was aimed at achieving a transfer in the vicinity of the near-infrared window. Hence, cresyl violet was chosen (λ_ex_ 602 nm, λ_em_ 620 nm) because its absorption bands fall in this region and its absorption spectrum overlaps well with the emission spectrum of rhodamine-101. Not only do cresyl violet excitation and emission bands seat nearby the near-infrared window (the so-called transparency window of biological tissues or optical/therapeutical window)^28^, they also seat near the edge of the CR emission spectrum. Despite being far from the CR emission peak a single population of cresyl violet could still undergo CRET with a resulting fluorescence emission around 620–650 nm (Fig. 3C, purple line). Upon addition of rhodamine-101 to the mixture, the same emission band resulting from a CRET-FRET process was observed (Fig. 3C, red line). Now when a third fluorophore was added (rhodamine-6G), the same emission band at 620–650 nm was still observed that is the result from a CRET-FRET-FRET process. However, the FRET process did not appear to be quantitative here because a small residual fluorescence emission was still observed that corresponds to the emission band of rhodamine-6G (a small bump within 550–560 nm, Fig. 3C, blue line). Overall the intensity of the signal in the 600–750 nm window, which results from the fluorescence of cresyl violet, is increased upon addition of the rhodamines. Such transfers correcting spectral outputs could also be represented as the form of CRET ratios per activity (Fig. 3B’, Table 2).

**Figure 4:**
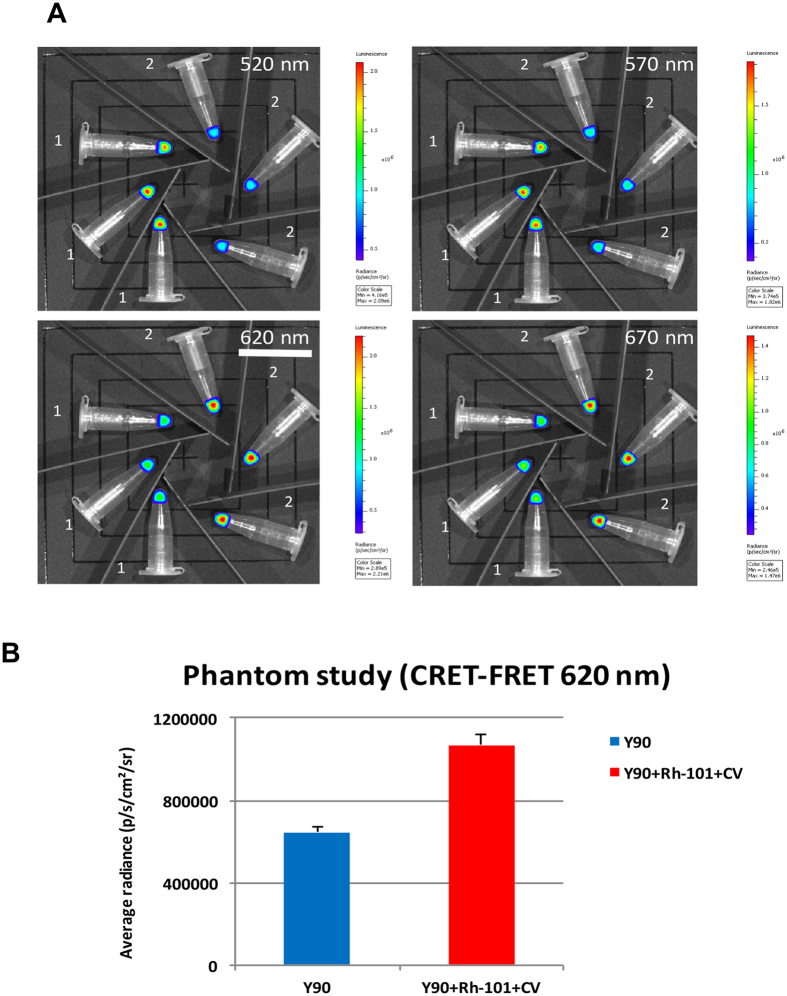
CRET-FRET on Phantoms (in triplicate) using IVIS optical imager. (**A**) sets of Eppendorf tubes filled with [^90^Y]-YCl_3_ only (tubes 1, left) *vs* sets of eppendorf tubes filled with [^90^Y]-YCl_3_ plus a ***binary*** fluorophore mixture Rh-101 + CV (tubes 2, right). Images collected using various filters (520 nm, 570 nm, 620 nm, and 670 nm): 620 nm filter is underlined and corresponds to the region of the spectrum where the radiance appears to be nearly doubled compared to that where CR is alone (it is the emission band of the last fluorophore of the cascade). (**B**) Measurement (bar graph) of the average radiance in the 610–630 nm window (620 nm filter) with solutions containing CR-emitting [^90^Y]-YCl_3_ only (in blue, left), or CR-emitting [^90^Y]-YCl_3_ mixed with a ***binary*** fluorophore mixture (Rh-101 + CV) that results in a nearly two-fold radiance boost (in red, right).

**Figure 5:**
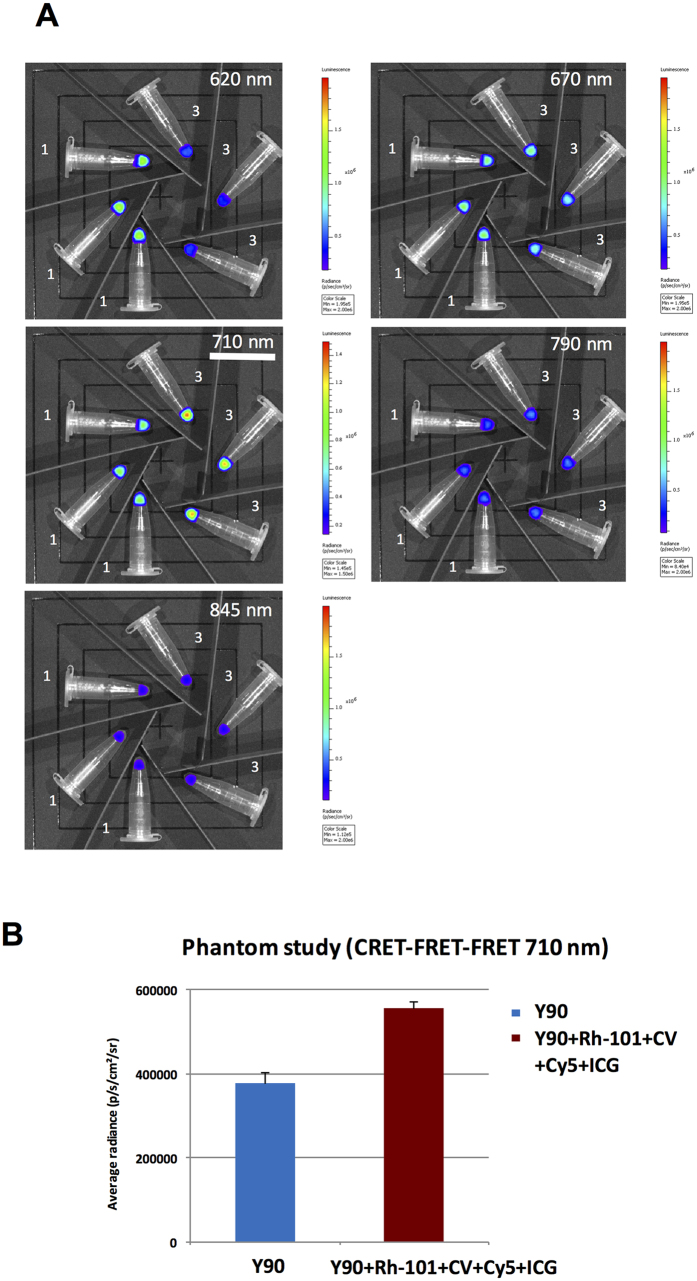
CRET-FRET-FRET on Phantoms (in triplicate) using IVIS optical imager. (**A**) sets of Eppendorf tubes filled with [^90^Y]-YCl_3_ only (tubes 1, left) *vs* sets of eppendorf tubes filled with [^90^Y]-YCl_3_ plus a ***quaternary*** fluorophore mixture Rh-101 + CV + Cy5 + ICG (tubes 3, right). Images collected using various filters (620 nm, 670 nm, 710 nm, 790 nm, and 845 nm): 710 nm filter is underlined and corresponds to the region of the spectrum where the radiance appears to be nearly doubled compared to that where CR is alone (the window is centered on the emission wavelength of one of the last fluorophore of the energy transfer cascade). (**B**) Measurement (bar graph) of the average radiance in the 700–720 nm window (710 nm filter) with solutions containing CR-emitting [^90^Y]-YCl_3_ only (blue bar, left), or CR-emitting [^90^Y]-YCl_3_ mixed with a ***quaternary*** mixture of fluorophores (Rh-101 + CV + Cy5 + ICG) that results in a nearly two-fold radiance boost (purple bar, right).

**Figure 6:**
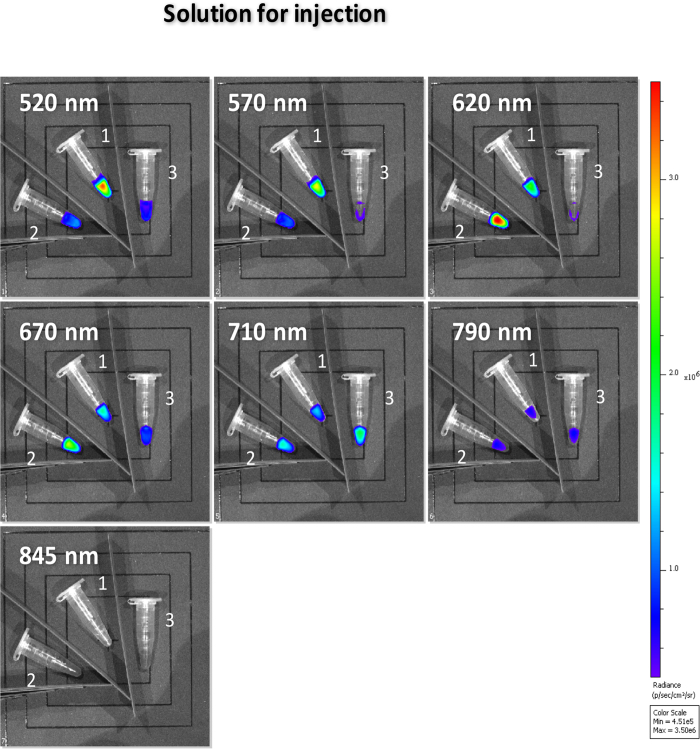
Images performed on IVIS optical imager of Eppendorf tubes using various filters (520 nm, 570 nm, 620 nm, 670 nm, 710 nm, 790 nm, and 845 nm). The tubes contain three solutions ready for injection (in mice) and made of [^90^Y]-YCl_3_ (1 MBq) mixed with either a) physiological serum only (tube 1, such a mixture is subject to **CR** only), b) or a ***binary*** fluorophore mixture, Rh-101 + CV (tube 2, such a mixture is subject to **CRET-FRET**), c) or a ***quaternary*** fluorophore mixture, Rh-101 + CV + Cy5 + ICG (tube 3, such a mixture is subject to **CRET-FRET-FRET** whereas CRET-FRET-FRET-FRET barely occurs).

**Figure 7:**
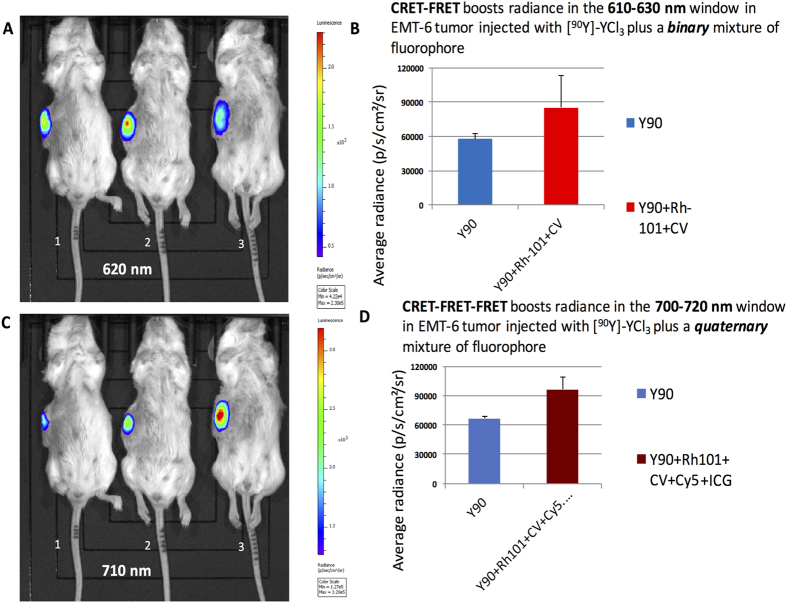
CRET-FRET and CRET-FRET-FRET processes *in vivo* in EMT6-tumor bearing mice (in triplicate) using IVIS optical imager. Mice were injected into the tumor with 50 μL of : [^90^Y]-YCl_3_ only (solution 1, mice 1), [^90^Y]-YCl_3_ and a ***binary***fluorophore mixture Rh-101 + CV (solution 2, mice 2), [^90^Y]-YCl_3_ and a ***quaternary***fluorophore mixture Rh-101 + CV + Cy5 + ICG (solution 3, mice 3). (**A**) Image collected using the 620 nm filter; (**B**) Measure of the average radiance in the 610–630 nm window (620 nm filter) with solutions containing CR-emitting [^90^Y]-YCl_3_ only (in blue, left), or CR-emitting [^90^Y]-YCl_3_ mixed with ***binary*** fluorophore mixture (Rh101 + CV) that results in a nearly **1.5**-fold radiance boost (in red, right). (**C**) images collected using the 710 nm filter; (**D**) Measure of the average radiance in the 700–720 nm window (710 nm filter) with solutions containing CR-emitting [^90^Y]-YCl_3_ only (in blue, left), or CR-emitting [^90^Y]-YCl_3_ mixed with ***quaternary*** fluorophore mixture (Rh-101 + CV + Cy5 + ICG) that results in a nearly **1.4**-fold radiance boost (in purple, right). *In vivo* studies were carried out in triplicate.

Next, other fluorophores emitting in the near-infrared such as, disulfonated cyanine-5 (Cy5) and indocyanine green (ICG) were examined to expand the cascade. No fluorescence emission (neither CRET nor CRET/FRET upon mixing Cy5 with Rh-101 and CV) could be detected with these fluorophores, even upon raising the radioactivity level. This may be explained a) eventually by a spectral overlap between CV and Cy5, and Cy5 and ICG that may not be as important as that with other fluorophores (Table 1), or more likely: b) because of the sensitivity of the spectrofluorimeter (Hamamatsu photomultiplier R928) that drops significantly beyond 650 nm, which may prevent the detection of low intensity signal (Fig. S1-A; this is unlike an IVIS optical imager, that was sensitive enough (Fig. S1-B) to detect such energy transfers up to Cy5 (Figs 4,5,6 and 7)); (c) or because of (b) together with the fact that the brightness of ICG is much smaller than that of other fluorophores (Table 1).

### CRET and CRET-FRET *ratios*

Next, a quantification of such transfers was attempted by determining the CRET ratios (Table 2). The method developed for determining CRET quantum efficiencies was adapted from that developed for FRET and BRET and later adapted by Piwnica-Worms (see Methods and windows in Fig. 1B)^15^,^20^,^30^,^31^,^32^. In a ***single*** population of fluorophore, we previously reported that CRET ratios drop when the fluorophore λ_max_ goes away from the CR maximum peak emission (Table 2)^20^. In ***binary*** systems, such as fluorescein/rhodamine 6G (Fig. 2B), the calculated CRET ratio per unit of radioactivity indicates that CRET-FRET (0.13) is barely less efficient than CRET alone (0.16) (Table 2, entries a-c). It concurs with the fact that the intensity of the emission peak in the mixture of both fluorophores is not higher than that of the single fluorophore (for the same amount of fluorophore in the cell). However, with another binary system, such as rhodamine-6G/rhodamine-101 (Fig. 2C) CRET ratios per unit of radioactivity indicate that CRET-FRET (0.042) is twice as more efficient than CRET alone (0.029) (Table 2, entries b, d, e). In ***ternary*** systems, such as fluorescein/rhodamine-6G/rhodamine-101 (Fig. 3B), the calculated CRET ratio per unit of radioactivity determined for CRET-FRET-FRET (0.027) is about the same as that of CRET alone (0.029, from rhodamine 101), but twice as less as that of CRET-FRET (0.042, from rhodamine-6G/rhodamine-101 binary system). This result shows that the CRET-FRET-FRET transfer with the actual set of fluorophores does not increase the intensity of fluorescence in higher wavelengths (as observed with rhodamine-101 and a mixture of rhodamine-101 + 6G) (Table 2, entries a, b, d, f). However, with another ternary system, such as rhodamine-6G/rhodamine-101/cresyl violet (Fig. 3C), CRET ratio per unit of radioactivity found with CRET-FRET-FRET (0.043) is slightly higher than that of CRET-FRET (0.037, with rhodamine-101/cresyl violet binary system), and 2.7 times higher as that of CRET alone (0.0162, with cresyl violet), i.e. signal amplification. Again, such results show that when a fluorophore λ_max_ lies too far away from the CR emission peak (i.e. the overlap between the two spectra is limited), its CRET ratio is low. However, if such a near-the-edge-of-CR absorbing fluorophore is now mixed with fluorophores the λ_max_ of which lies nearby the CR peak emission, subsequent CRET ratios seem to be raised. Overall CRET ratios at the acceptor were always equal or increased in CRET-FRET-FRET or CRET-FRET compared to CRET only.

## Phantom Studies on the optical imager

Phantom studies on the IVIS photon imager were performed in Eppendorf tubes filled with a solution (50 μL) of either [^90^Y]-YCl_3_ alone or [^90^Y]-YCl_3_ mixed with a given mixture of fluorophore. A series of filters were used (520, 570, 620, 670, 710, 790, and 845 nm): given the 10-nm bandwidth, the corresponding radiance measurements were achieved in 20-nm wide windows. Subsequent estimate of transfer efficiencies could be achieved, on a selected window, upon comparing the radiance resulting from CR alone (Eppendorf filled with CR-emitting radionuclides, only) with the radiance resulting from CR, CRET and FRET cascades (Eppendorf filled with both radionuclides and fluorophores).

### CRET-FRET (620 nm)

With the ***binary*** mixture (Rhodamine-101 (Rh-101) + Cresyl Violet (CV)) the same trend as that described on the spectrofluorimeter (Fig. 3C) was found on the imager (Fig. 4A). First, the radiance was measured for the Cherenkov Radiation only (i.e. tubes filled with [^90^Y]-YCl_3_ only, Fig. 4A, set of tubes 1, left) : it is strong in the low wavelengths, such as 520 nm and 570 nm, whereas it drops at higher wavelengths, such as 620 nm and 670 nm. This is in accordance with the profile of the CR emission spectrum measured by spectrofluorimetry (Fig. 1B)^15^,^17^,^20^. Second, the radiance profile is the other way around with a solution of [^90^Y]-YCl_3_ mixed with the ***binary*** mixture of fluorophores (Rh-101 + CV; Fig. 4A, set of tubes 2, right): in the 610–630 nm window the average radiance is **1.7** times as much with ^90^Y + Rh-101 + CV than with ^90^Y alone (Fig. 4B). Hence, data depicted in Fig. 4A,B are an additional evidence (beside the spectroscopic data provided in Fig. 3C), of the occurrence of CRET and CRET-FRET cascades leading to the fluorescence emission of CV, which results in an overall radiance boost by almost a two-fold factor in this narrow window (compared to the case of CR only). Note that a fluorophore “quenching effect” on CR is noticed in the 520 nm and 570 nm windows: this may simply be the result of a significant absorption/consumption of CR by the fluorophores (the main absorption bands of which lies precisely in this region) resulting in a significant drop of radiance (subsequent CRET and FRET transfers occur beyond these selected windows).

### CRET-FRET-FRET (>710 nm)

A similar experiment was conducted using a ***quaternary***mixture made of Rh101, CV, Cyanine 5 (Cy5), and Indocyanine Green (ICG). The quaternary mixture is basically the binary mixture (Rh101 plus CV, described above) to which near-infrared absorbing/emitting fluorophores have been added, such as Cyanine 5 (λ_abs_ = 675 nm, λ_em_ = 694 nm) and ICG (λ_abs_ = 805 nm; λ_em_ = 835 nm). The new dyes were chosen because: a) of a good spectral overlap between each other and between the last component of the *binary* mixture, b) their emission in the near-IR which is relevant to imaging of biological tissues, c) and FDA approval of ICG. First, the radiance evolution measured for the Cherenkov Radiation only (i.e. tubes filled with [^90^Y]-YCl_3_ only, Fig. 5A, set of tubes 1, left) was the same as that measured before (Fig. 4A): CR is very intense in the 520–570 nm window (data not shown), its intensity drops significantly around 620–710 nm, and becomes definitely less significant in the 790–850 nm window. Second, the radiance profile is the other way around with a solution of [^90^Y]-YCl_3_ mixed with the ***quaternary*** mixture of fluorophores (Rh-101 + CV + Cy5 + ICG; Fig. 5A, sets of tubes 3, right). As shown with the *binary* mixture, CR quenching occurs at low wavelengths, possibly as the result of a significant CR absorption by Rh-101 and CV fluorophores. Such a quenching phenomenon is now observed up to 620 nm: Rh-101 and CV do absorb CR (as a result it leads to a significant radiance drop) while their emissions are no longer observed at 620 nm because subsequent energy transfer to the next fluorophores do occur. Hence, further towards the red region of the spectrum (670 nm and 710 nm filters), and as a result of such transfers, subsequent luminescence turn-on occurred (Fig. 5A, tubes 3, right): in the 700–720 nm window the average radiance is **1.5** as much with [^90^Y]-YCl_3_ mixed with Rh-101 + CV + Cy5 + ICG (Fig. 5B, purple bar, right) than with [^90^Y]-YCl_3_ alone (Fig. 5B, blue bar, left). This is an indication that energy transfers, i.e. CRET/CRET-FRET-FRET cascade processes occurred efficiently at least up to Cy5 (λ_em_ = 694 nm) and that further cascade to ICG does not seem to occur efficiently (Fig. 5A, 790 nm and 850 nm windows show no radiance boost).

## ***In vivo*** Studies on the Optical Imager

### Solutions before injection

Based on the previous results, three working solutions were prepared for *in vivo* studies. These mixtures were examined on the optical imager (Fig. 6) prior injection to mice, using the same set of filters as that described above (520 nm, 570 nm, 620 nm, 670 nm, 710 nm, 790 nm, 845 nm). They were either a solution of [^90^Y]-YCl_3_ alone in physiological serum (solution 1, tube 1, center) or a solution made of [^90^Y]-YCl_3_ plus a given set of fluorophores (that was previously studied on the spectrofluorimeter and the imager on phantoms, solutions 2–3, tubes 2–3). The chosen mixture of fluorophores was either ***binary*** (solution 2, tube 2, left: Rh-101 + CV; *see* Figs 3 and 4A,B) or ***quaternary*** (solution 3, tube 3, right: Rh-101 + CV + Cy5 + ICG; *see* Fig. 5A,B). Prior injection on mice, all three solutions analyzed on the optical imager showed the same trend (Fig. 6) as that shown before (Fig. 5), with nearly two-fold radiance boosts either: a) on the 610–630 nm window (620 nm filter) with the ***binary*** mixture of fluorophores, or b) on the 710–730 nm window (720 nm filter) with the ***quaternary***mixture of fluorophores.

### *In vivo* studies

Injection of a working mixture (50 μL) into tumors (injected about 1 cm deep) was achieved in three EMT6 breast carcinoma bearing BALB-c mice in triplicate. The first mice were injected with solution 1 ([^90^Y]-YCl_3_ alone in physiological serum), the second mice was injected with solution 2 ([^90^Y]-YCl_3_ in physiological serum mixed with ***binary***fluorophore mixture), and the third mice was injected with solution 3 ([^90^Y]-YCl_3_ alone in physiological serum mixed with ***quaternary***fluorophore mixture). Subsequent imaging studies were performed on the IVIS optical imager with the three mice at once. The filters selected for *in vivo* studies were the same as that used for phantom studies (Figs 4,5 and 6), i.e. 620 nm (Fig. 7A,B) or 710 nm (Fig. 7C,D). The same variations in radiance (as that shown in Figs 4,5 and 6) were observed with each chosen pair (filter/fluorophore mixture). First, it showed that in the 610–630 nm window (Fig. 7A,B) the radiance is **1.5**-fold greater in tumor injected with solution 2 ([^90^Y]-YCl_3_ plus the ***binary*** fluorophore mixture (that is the result of a CRET-FRET process) in mice 2) than that injected with solutions 1 ([^90^Y]-YCl_3_ alone, resulting in CR emission only, in mice 1) or solution 3 ([^90^Y]-YCl_3_ plus the ***quaternary*** fluorophore mixtures, resulting in CR alone and emission of fluorophores occurring beyond the selected window, in mice 3). Imaging studies achieved upon selecting the 710 nm filter (Fig. 7C) showed that the signal is **1.4**-fold greater in tumor injected with solution 3 ([^90^Y]-YCl_3_ plus the ***quaternary*** fluorophore mixture (that is the result of a CRET-FRET-FRET process), in mice 3) than that injected with the other mixtures, such as solution 1 ([^90^Y]-YCl_3_, CR alone, in mice 1) or solution 2 ([^90^Y]-YCl_3_, CR alone plus the ***binary*** set of fluorophores the absorbance maxima of which are below the window of interest), in mice 2). These *in vivo* results are in accordance with the results on phantoms, albeit with slight attenuation that may be the result of light interaction (absorption and scattering) with tumor tissues.

In summary, this study showed that mixing a Cherenkov Radiation (CR) emitter, such as Yttrium-90, with several fluorophores that have λ_ex_/λ_em_ couples matching each other (good spectral overlap) led to a cascade of energy transfers. The first type of transfer, Cherenkov Radiation Energy Transfer (CRET) occurs from the radionuclide to the fluorophore. The second type of transfer is the Förster Resonance Energy Transfer (FRET) that takes place from one fluorophore to another. Herein we have reported the FRET-type transfer occurring subsequently to CRET and between up to three fluorophores present in the system. Hence the overall process, called CRET-FRET and CRET-multiFRET (or Cherenkov-induced FRET or CRET-FRET-FRET-FRET) appears to be the first of its kind to be reported in the literature. Indication regarding the efficiency/yields of transfers are given from the calculated CRET ratios. Fluorophores, such as cresyl violet, that are both near-IR-emitting but also near-the-edge of CR spectrum absorbing, get low CRET ratios (low fluorescence emission), precisely because its absorption spectrum and the CR emission spectrum do not overlap much. This was expected based on our previous report^20^. However, loading additional fluorophores the emission bands of which overlap significantly with the maximum emission peak of CR and the absorption bands of such near-IR emitting cresyl violet, could significantly increase the emission of the latter, and hence the overall CRET-FRET ratio. Such a result is significant because it shows that blue-weighted CR (300–600 nm) activation of molecular luminescent platforms : a) gives rise to a fluorescence emission as far as **710** nm, b) it occurs with some amplification of the signal due to FRET: EMT6-tumor bearing mice injected with a radionuclide together with a mixture of fluorophore (with good spectral overlap) was shown to have a two-fold radiance boost (on a window centered at the wavelength corresponding to the last fluorophore of the cascade) compared to a tumor injected with the radionuclide only. These data are in accordance not only with studies on phantoms, but also with spectrofluorimetric studies. This study using organic fluorophores is an important proof-of-concept highly relevant to biological studies because tissues are opaque in the blue region of the spectrum where the CR maximum emission peak lies (note that it echoes previous studies with inorganic nanoparticles, such as quantum dots, albeit not involving FRET)^15^,^16^,^17^,^18^,^21^,^22^,^23^. In the near infrared window, the benefit of such a CRET-(FRET)_n_/NIR strategy is obvious because it led to a radiance boost compared with the weak radiance of CR in that window. However, the benefit of such a NIR strategy has a photon cost (efficiencies of the CRET/FRET cascade), which may be compensated with the lifting of opacity (Fig. 1) in the NIR region (optical window). Hence, “standard” CLI relies on blue-weighted CR, where there is “ample” supply of photons a large portion of which will be lost upon crossing biological tissues (Fig. 1C). In the CRET-multiFRET approach, i.e. a strategy to achieve a redshift of CR, a portion of photons is lost throughout the cascade of transfers before reaching the 700–720 nm window, but subsequent crossing of biological tissues from such a window (the so-called therapeutic/optical window) will be much higher than in the blue/UV region (Fig. 1C). As a conclusion the radiance in the near-infrared window from a mixture of ^90^Y and several fluorophores (CRET-FRET) remains higher than that resulting from ^90^Y alone (CR only). Such an approach reported in this study showed how to red-shift a significant portion of blue weighted CR, into the near-IR region of the spectrum (**710** nm), hence to increase the radiance in this window. Although this study remains a proof-of-concept *per se*, it appears that these findings might be of major importance to perform a theranostic approach for cancer management.

## Methods

### Radioactive fluorescence measurements

Cerenkov Radiation Energy Transfer (CRET) Fluorescence measurements were performed on a Agilent Cary Eclipse (sensitivity: *a*) signal-to-noise measurements of Raman band of water 1/700 (detection limit 1 pM fluorescein); *b*) theoretical detection limit: 1 pmol of fluorescein) in quartz cuvettes 1 × 1 × 3 cm (1 cm path). The method used was the following: 400 μL of fluorophore (10^−3^ M, 5 × 10^−4^ M, 10^−4^ M in a water-miscible solvent) was mixed with a solution of the radioactive species, [^90^Y]-YCl_3_ (activity 6–10 MBq, Figs 2 and 3), supplied by Perkin-Elmer (USA) the volume of which was adapted depending on the desired radioactivity level to be introduced in the cuvette (i.e. the radionuclide: fluorophore molar ratio is small)^20^. Subsequent addition of a saline solution (0.9% NaCl) was achieved to reach an overall volume of 1 mL. Hence, such studies required significant concentrations in fluorophores, which may induce some stacking and affect their optical properties, possibly resulting in the 20 nm bathochromic shift noticed in the fluorescence emission spectra. Measurement parameters were: bioluminescence mode, gate time of 10 s, 20 nm emission slit, 3 nm of data interval, Stavinsky smoothing (factor 5). Radioactivity was measured with a dose calibrator (MEDI 405, Medisystem, France). Baseline curves were found to be pretty dependent upon the volume of saline solution added and the nature of the solvent in the fluorophore solution. That is why, to be comparable, curves were adjusted to a same value at 700 nm by substraction with an adapted constant value^20^.

### CRET and CRET-FRET *ratios*

The method developed for determining CRET quantum efficiency was adapted from that developed for FRET and BRET and later adapted by Piwnica-Worms Table 2^15^,^20^,^30^,^31^,^32^. We define the CRET ratio as the subtraction of two quotients of light (one related to both CR and CRET, another one to CR only)(eqn 1) based on the identification of two spectral windows in the spectrum (window A (X1 − X2) corresponds to the main emission of the fluorophore, whereas windows B1 and B2 (Y1 − X1 and X2 − Y2, respectively) are peripheral and correspond to the residual emissions, see Fig. 1B). The first quotient corresponds to the overall light (the sum of both CR and CRET) detected within a narrow spectral window A (X1 − X2, that is exclusively centred on the fluorophore emission) divided by the overall light (the sum of both CR and CRET) detected on a larger spectral window (Y1 − Y2, which comprises Y1 − X1 + X1 − X2 + X2 − Y1). The second quotient is pretty much alike the first one: while it also involves the same set of spectral windows (X1 − X2 and Y1 − Y2), however it only considers CR (and no CRET from the fluorophore).


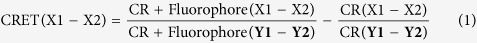


### Phantoms and *in vivo* studies with optical imaging

#### Radioactive Stock solutions

The radioactive stock solution was based on the following mix (% of total volume): 50% of [^90^Y]-YCl_3_ (370 MBq at calibration, Perkin-Elmer, USA), 25% EDTA (50 mM in AcONH_4_, 0.1 M) and 25% of isotonic NaCl (0.15 M). Working solution of the ***binary*** fluorophore mixture contains rhodamine-101 and cresyl violet in solution in PBS (final concentration of both fluorophores: 0.1 mM) plus 1 MBq [^90^Y]-YCl_3_. Working solution of the ***quaternary*** fluorophore contains rhodamine-101, cresyl violet, cyanine 5.19 and IndoCyanine Green (ICG, Infracyanine^®^, Laboratoires SERB, France) in solution in PBS (final concentration of each four fluorophores: 0.1 mM) plus 1 MBq [^90^Y]-YCl_3_.

#### Phantoms

Phantoms studies were carried out on Eppendorf tubes filled with 50 μL of working solutions, *i.e* either *binary* or *quaternary* fluorophore solution (0.1 mM final concentration for each fluorophore in PBS) containing [^90^Y]-YCl_3_ (1 MBq).

#### Solutions for intratumor injection

Syringes filled with either [^90^Y]-YCl_3_ (1 MBq) in isotonic NaCl or a mixture of fluorophores (*binary* or *quaternary*, 0.1 mM final concentration for each fluorophore in PBS) plus [^90^Y]-YCl_3_ (1 MBq) were prepared prior injections into the tumor.

#### Mice

Mice were maintained in ventilated housing units under controlled conditions of temperature (22 ± 2 °C), photoperiod (12 h light/12 h dark) with free access to food and drink. Tumors were induced by subcutaneous injection of 1 × 10^6^ EMT6 cells in 200 μL of RPMI 1640 into the right flank of BALB/c mice (BALB/cByJ, Charles River, France). EMT6 (ATCC) cell line was established from a transplantable murine mammary carcinoma that arose in a BALB/cCRGL mouse after implantation of a hyperplastic mammary alveolar nodule^33^. After tumor growth and prior to *in vivo* imaging, mice were divided in 3 experimental groups (n = 3 per group) in order to receive one intratumor injection of either [^90^Y]-YCl_3_ (1 MBq) in isotonic NaCl or the mixture of fluorophores (binary or quaternary, 0.1 mM final concentration for each fluorophore in PBS) containing [^90^Y]-YCl_3_ (1 MBq). Then, mice were anaesthetized with isoflurane (3–4% in oxygen) then euthanized with intraperitoneal pentobarbital overdose (160 mg/kg) and subsequently injected within their tumors with 50 μL of the *ad hoc* radioactive solution.

#### Optical imaging

Optical imaging was performed with an IVIS Lumina III system (Perkin Elmer, USA) in bioluminescence mode, *i.e* without any excitation light. Optical signal was normalized to average radiance expressed in photons per second per centimeter square per steradian (p/s/cm^2^/sr). Wavelength resolved spectral imaging was carried out using a set of narrow bands emission filters: 520, 570, 620, 670, 710, 790 and 845 nm. Depending on the experiment the filter of choice were selected, either all to get a complete view (Fig. 6) or narrowed to a few filters (Fig. 7). For the phantom and mice studies the acquisitions were performed over wavelength (emission) ranging from 520 to 845 nm and acquisition parameters were as follows: binning factor 8; field of view 12.5 cm, F number 1, exposure time from 10 to 60 seconds. Images were acquired and analyzed using Living Image 4.5.2 software (Perkin Elmer, USA).

### Name of the institutions or licensing bodies that approved the experimental protocols

#### Nuclear safety

(a) Safety rules and protocols were set at and approved by the *Centre George-François Leclerc* (CGFL) and applied at CGFL Preclinical Imaging Platform; (b) The protocols strictly comply with the regulations governing radiopharmaceuticals^34^ and standards of the *French Nuclear Safety Agency* (ASN) that approves CGFL protocols.

#### In vivo studies

All animal studies were conducted at and approved by the Centre George François Leclerc (CGFL) in accordance with the relevant guidelines and legislation on the use of laboratory animals (directive 2010/63/EU) and were approved by accredited *Ethical Committees* (Oncomet n°91 and C2ea Grand Campus n°105) and *French Ministry of Higher Education and Research*.

#### Radioactive fluorescence studies

Protocols comply with standards of fluorescence studies set at ICMUB Institute and revisited at CGFL on radioactive materials^20^.

## Additional Information

**How to cite this article:** Bernhard, Y. *et al*. Redshifted Cherenkov Radiation for *in vivo* Imaging: Coupling Cherenkov Radiation Energy Transfer to multiple Förster Resonance Energy Transfers. *Sci. Rep.* **7**, 45063; doi: 10.1038/srep45063 (2017).

**Publisher's note:** Springer Nature remains neutral with regard to jurisdictional claims in published maps and institutional affiliations.

## Supplementary Material

Supplementary Information

## Figures and Tables

**Table 1:** Selected fluorophores for CRET and CRET-multiFRET studies.

	Fluorescein	Rhodamine 6G	Rhodamine 101	Cresyl Violet	Cyanine 5	Indocyanine Green
λ_abs_/λ_em_ (nm)	490/514	530/552	576/600	602/620	675/695	805/835
Φ_F_ x ε	73,000	109,000	95,000	44,820	67,000	1,763

**Table 2:** CRET Ratios, activities, and ratios for a mixture containing one (entries a, b, d, g), two (entries c, e, h), or three fluorophores (entries f, i).

Entry	Fluorophore mixture (spectral windows B1 and B2)*	CRET Ratio	Activity (MBq)	CRET Ratio per activity (MBq^−1^)
a	Fluorescein (400–475/600–700 nm)	1.97	7.31	0.27
b	Rhodamine-6G (400–525/650–700 nm)	1.09	6.78	0.16
c	Fluorescein + Rhodamine-6G (400–525/650–700 nm)	0.91	6.89	0.13
d	Rhodamine-101 (400–600/700–700 nm)	0.19	6.61	0.029
e	Rhodamine-6G + 101 (400–600/700–700 nm)	0.33	7.80	0.042
f	Fluorescein + Rhodamine-6G + 101 (400–600/700–700 nm)	0.18	6.72	0.027
g	Cresyl violet	0.15	9.26	0.0162
h	Cresyl violet + Rhodamine-101	0.37	10.13	0.0365
I	Cresyl violet + Rhodamine-6G + 101	0.44	10.15	0.0433
