# Supplementary material for: Redshifted Cherenkov Radiation for in vivo Imaging: Coupling Cherenkov Radiation Energy Transfer to multiple Förster Resonance Energy Transfers
Source: Sci Rep. 2017 Mar 24;7:45063. doi: 10.1038/srep45063 (PMC5364485; doi:10.1038/srep45063)
Supplement: Supplementary Information [file srep45063-s1.pdf]

Supporting Information

Redshifted Cherenkov Radiation for *in vivo* Imaging: Coupling Cherenkov Radiation Energy Transfer to multiple Förster Resonance Energy Transfers .

Yann Bernhard,[a] Bertrand Collin,[a,b] and Richard A. Decréau [a]

Richard.Decreau@u-bourgogne.fr

Address

[a] ICMUB Institute, UBFC - University Burgundy Franche Comté (University of Dijon), 9 avenue Alain Savary, 21078 Dijon (France); [b] CGFL Cancer Research Center, 1 rue du Professeur Marion, 21079 Dijon (France)

Sensitivity of the spectrofluorimeter *vs* that of the optical imager

Spectrofluorimeter (Agilent Cary Eclipse)

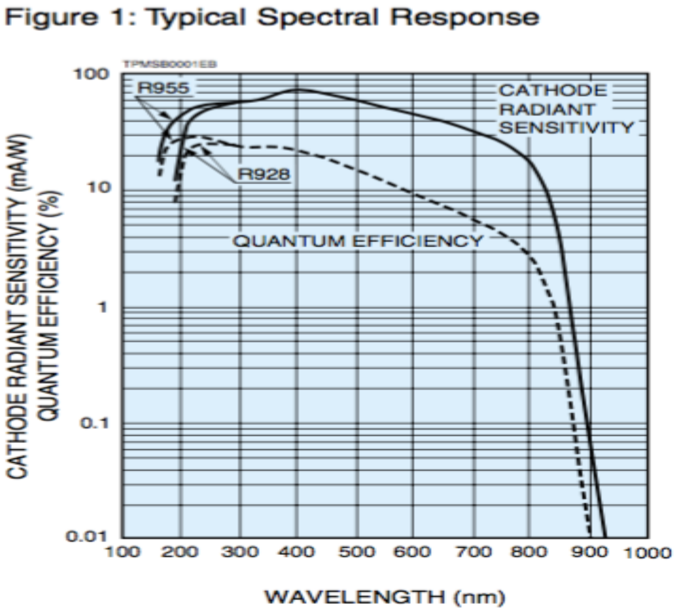

With permission from Hamamatsu and Agilent Cary Eclipse

IVIS Lumina III (Perkin Elmer)

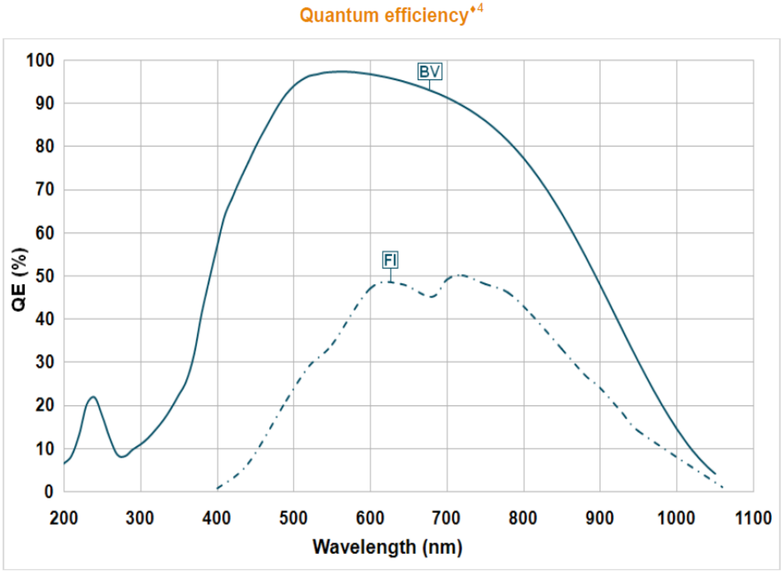

With permission from Perkin Elmer
